# Translating One Health knowledge across different institutional and political contexts in Europe

**DOI:** 10.1186/s42522-022-00074-x

**Published:** 2023-01-31

**Authors:** Sarah Humboldt-Dachroeden

**Affiliations:** Department of Social Sciences and Business, Roskilde University, Roskilde, Denmark

**Keywords:** One health, Knowledge translation, Networks, Leadership, Political attention, Research communication

## Abstract

**Background:**

Implementing a One Health approach is complex. It demands engaging different sectors and actors in the promotion and protection of human, animal and environmental health. A key challenge for successfully implementing the One Health approach are knowledge translation processes among scientists and policy-makers.

**Methods:**

An online survey reached 104 experts from 23 European countries, working at national agencies or institutes, universities, ministries, non-governmental organisations (World Health Organization, World Organisation for Animal Health), and European Union (EU) agencies. Qualitative and quantitative analyses were conducted to describe experts’ perceptions.

**Results:**

This study indicated a lack of networks among scientists and between scientists and policy-makers. Relations of scientists and policy-makers were perceived as challenging due to different interests and priorities, leading to difficulties in reaching political attention for One Health topics. It also highlighted a favoured attention to some One Health topics (e.g. antimicrobial resistance) as opposed to others (e.g. environmental issues). Important international actors to push One Health policies forward were the Quadripartite organisations and EU agencies. National actors (government agencies, national research institutes, universities) were on average perceived to be more important than international actors due to their roles and influences. Factors influencing the knowledge translation process were the different languages spoken by scientists as well as politicians, and an equivocal understanding of the One Health approach.

**Conclusion:**

The study shows the importance of leadership to establish interdisciplinary networks and to problematise One Health issues with clear scope and targets. This will help to link knowledge to needs and capabilities of policy-makers. Establishing strong relationships among national and international actors can encourage networks and raise awareness of the One Health approach to policy-makers. Lastly, promoting research communication skills of scientists can provide a valuable tool to reach policy-makers to enhance attention to One Health topics.

**Supplementary Information:**

The online version contains supplementary material available at 10.1186/s42522-022-00074-x.

## Background

One Health is an approach that connects public health, veterinary and environmental sectors. It aims to tackle societal issues, such as threats to ecosystems, zoonotic diseases, which are diseases that spread from animals to humans and vice versa, or antimicrobial resistance (AMR), which happens when microorganisms develop and become resistant to conventional treatments that are used to treat infectious diseases (among other treatments this includes antibiotics). To achieve this, the approach is based on collaborations, communication and coordination across the sectors and relevant actors [1]. Collaboration is a key aspect for the integration of different disciplines and expertise to enable knowledge sharing [2]. Implementing a One Health approach can lead to enhanced disease control, biosecurity procedures, and can identify opportunities for health promotion and risk mitigation on the human-animal-environment interface [3, 4]. It is a multifaceted approach entailing the integration of different sectors (e.g. public health, medical, animal health, environment, food safety) and actors (e.g. bureaucrats, politicians, scientists, health care providers, industry, public) who follow their own agendas and priorities [5]. The implementation of One Health activities broadens the scope of study designs due to the engagement of multiple sectors, and the different types of knowledge from various sources, such as scientific evidence from scientists of different disciplines [3, 6].

However, the literature describes silos between the sectors and how they present a challenge for implementing One Health activities [2, 7, 8]. Often the silos arise due to difficulties in collaborating, communicating and translating information across sectors, disciplines as well as outside one’s own epistemic community [7]. This may lead to a lack of political awareness and hence, resources and funding of One Health initiatives [2]. A key premise for enabling the implementation of the One Health approach is the translation of knowledge across research disciplines, and from scientists to policy-makers [8, 9]. However, little is known about the knowledge translation processes among scientists and policy-makers, such as bureaucrats and politicians. Knowledge translation processes take part among different actors and sectors, they can facilitate the coordination of One Health activities, connect actors promoting collaboration and access of data [10]. Investigating the knowledge translation process for the One Health approach among scientists and policy-makers can give insight into obstacles for implementing the One Health approach.

The aim of this study is therefore to comprehend institutional and political structures that enable the knowledge translation process for the One Health approach. This is one of the few studies that examines some of the knowledge translation challenges that impede the implementation of the One Health approach by including experts’ perceptions of institutional and political challenges.

The theoretical basis of the study is the knowledge transfer model to shed light on the translation of knowledge (scientific evidence) from the source (scientists) to the receiver (policy-makers) [11]. This study will especially investigate networks and relations of the source and receiver in terms of transforming, associating, and applying knowledge, and provides insight into some influencing factors. Transforming knowledge is the process of making knowledge useful for the receiver, and associating it entails linking it to policy-makers’ needs and capabilities. Transformed and associated knowledge can then be applied by the receiver to create value [11]. Influencing factors are elements that can affect networks, actors, their relations and thus they can affect the process of transferring knowledge either positively, enhancing the process, or negatively, impeding the process [11, 12]. This paper limits itself to the influencing factors of capabilities and skills to assess potential avenues that enhance positive and avoid negative influences for the knowledge transfer process. The dimensions (networks, relations and influencing factors) will structure the analysis and provide an insight into the knowledge translation processes between scientists and policy-makers.

This study is based on an online survey about the governance of One Health. It involved 104 scientists, experts and policy-makers from the sectors of public and veterinary health, environment and food. The paper finds that there are some unsatisfied opportunities and identifies three areas in which to improve the knowledge translation process of One Health activities: Networks, relations of scientists and policy-makers, and influencing factors. The results indicate that the uptake of the One Health approach within European agencies and institutes is insufficient, impeding comprehensive and cross-sectoral considerations of health on the human-animal-environment interface. This study demonstrates some of the constrains that can be used as lessons learned and inspire future planning, designing and implementing of One Health activities.

## Methods

The study employed a mix of quantitative and qualitative data from the survey to inform the three dimensions: networks, relations and influencing factors.

### Online survey

An online survey was created with version 12.9 of SurveyExact by Rambøll Management Consulting. The questionnaire contains 17 questions categorised under the sections Demographics, Experience with One Health, Science to Policy, Coordination of One Health, End (see Additional file 1). The survey was anonymous and no sensitive nor personal information was gathered.

Prior to launching the survey, the questionnaire was examined by four colleagues from the fields of social and veterinary sciences, which optimised the understanding, language and structure of the questions. Subsequently, a pilot study was conducted with 21 scientists in the fields of public health, veterinary, food and environmental sectors. The pilot study was performed over ten days in March 2021 and was evaluated for coherence, objectiveness and relevance. This led to refine demographic questions, explanatory and technical aspects, and clarifications of content and structure, which strengthened construct validity of the survey. The survey was open from March to July 2021 and completed by 104 experts from 23 European countries, see Table 1. Survey respondents were selected based on a purposive sampling strategy. The study was part of the (European Union) EU Horizon 2020 project One Health European Joint Programme (OHEJP), which contains projects working on One Health topics. The OHEJP provided access to experts in the areas of medicine, public health, veterinary, environment and food sciences in 22 EU countries. The survey was distributed via mailing lists to OHEJP members. Additional own searches located more survey respondents working in relevant sectors. The response rate of the survey was 46.8%.

The survey respondents specified their workplaces and areas of work (Table 1). The survey included 68 natural and 13 social scientists (plus 23 respondents who did not specify their background). Respondents with a background in veterinary sciences (*n* = 45), biology (*n* = 13), agriculture (*n* = 5), physics (*n* = 2), environmental science (*n* = 2), and medicine (*n* = 1) were categorised into the natural sciences, while respondents with a background in public health (*n* = 6), law (*n* = 3), social science (*n* = 2) and public administration (*n* = 2) were categorised into the social sciences. The responses represented the respondents’ subjective perspective that they obtained through their work and the country they live in.

**Table 1:** Characteristics of respondents

	Countries	[n]	Workplace				[n]	Areas^d^	[n]
Western Europe	United Kingdom	10	Veterinary institute				18	Zoonotic diseases	73
	Germany	9	Public health institute				17	Antimicrobial resistance	63
	France	8	University				12	Food safety	58
	The Netherlands	7	Food institute				12	Disease surveillance	56
	Belgium	8	Ministry (Ministries of Agriculture; Health; Education and Research)				7	Disease prevention & preparedness	56
	Austria	4	NGO (WHO, WOAH, ICARS^a^)				5	Food security	24
	Switzerland	3	Interdisciplinary research institutes:					Environmental contamination	23
			Vet	Food	Env	Agri			
			x	x			4		
				x	x	x	4		
			x	x	x		4^b^		
			x	x	x	x	3		
			x			x	2		
						x	2		
	Ireland	2	EU agency (EFSA, EMA, EEA^c^)				4	Climate change	17
Nordic Countries	Sweden	10	Funding institute				1	Biodiversity	13
	Denmark	8	Museum (Natural history)				1	Other	19
	Norway	3	N/A				8		
	Finland	3							
Southern Europe	Italy	9							
	Portugal	6							
	Spain	1							
Eastern Europe	Hungary	2							
	Lithuania	2							
	Bulgaria	1							
	Czech Republic	1							
	Estonia	1							
	Latvia	1							
	Poland	1							
	Romania	1							
	N/A	3							
	Total	104					104		402
	Countries: 23								
	EU countries: 20								
	European countries: 3								

^a^*WHO* World Health Organization, *WOAH* World Organisation for Animal Health, *ICARS* International Centre for Antimicrobial Resistance Solutions

^b^One research institute also includes public health services

^c^*EFSA* European Food Safety Authority, *EMA* European Medicines Agency, *EEA* European Environment Agency

^d^Areas respondents work with – multiple responses were possible

### Analytical approach

The analysis of the open-ended questions was conducted via the software NVivo Pro (version 12). A content analysis was conducted, and seven themes were established: Attention; Government & governance structures; Networks & activities; Roles; Influences; Interests & priorities; Scientific language. This followed inductive reasoning, finding patterns within the respondents’ statements that allowed for the above-mentioned categorisation into the themes, which were then related to concepts (networks, relations, influencing factors) of the knowledge transfer model. These themes were reviewed to ensure consistent and appropriate categorisation of codes into the themes. The following three sub-chapters in the result section encompass these themes. Each of the 104 respondents were assigned a number, which allowed to connect them to their statements and survey choices. These numbers identify the participants (P) and their workplace when quoted in the Results section (e.g. (P15 – University).

The closed questions allowed for a quantitative analysis that was conducted via the IBM SPSS Software (version 27). Descriptive statistics of respondents’ characteristics in terms of respondents’ countries, workplace and areas of work were examined. Some measures of central tendencies were conducted in relation to respondents’ perception of challenges for the implementation of One Health; respondents’ categorisation of communication, attention and translation issues between scientists and policy-makers; and respondents’ ranking of importance of international and national actors.

Two independent t-tests were conducted to compare means across sub-groups of the population to investigate whether (1) coming from Nordic countries, western, southern and eastern Europe (see Table 1 for categorisation); or (2) having a background in social or natural sciences showed differences from one another. The categorisation of educational backgrounds into natural and social sciences is broad and limits itself in presenting the variability of the disciplines, including their unique ways in addressing and approaching issues. Nevertheless, this categorisation allows the comparison of two groups that have fundamentally different educations but both work with One Health.

## Results

### Networks – (dis-)connections between ministries

Establishing networks, for example across ministries can be challenging due to increased compartmentalisation [13]. This was also perceived by ministries dealing with the One Health approach, as one survey respondent put it: “Policy-makers are sitting in different ministries. Much depends [on] how good the communication and collaboration between the ministries [is] in reaching the common understanding” (P15 – University). Especially the collaboration across ministries, which deal with topics on the human-animal-environment interface was perceived to be more challenging and indicates disciplinary silos and a lack of networks, see Table 2.

**Table 2:** Respondents’ perception of challenges for the implementation of One Health^a^

	Number of respondents (n)	Answers (%)^b^
Lack of funding	53	20
Lack of collaboration between ministries	49	18
Lack of political awareness	43	16
Inadequate governance/leadership	36	13
Lack of education and training	20	7
Lack of communication between institutes	16	6
Lack of collaboration between institutes	16	6
Lack of willingness	8	3
Confusing legislation	8	3
Lack of guidance	4	1
Other	18	7
*Total*: *Total number of responses*	100 271	

^a^Question refers to the respondents’ respective country

^b^Max. three choices

The main challenge identified by the respondents was a lack of funding, followed by structural and organisational issues, like the lack of collaboration across ministries, the lack of political awareness and the lack of governance/leadership, which were all among the top four challenges perceived by the survey respondents (Table 2). The organisation of networks on a ministerial level can provide a way in which information can be shared across sectors to ensure a more comprehensive perspective. To increase political awareness of the One Health issues, it was suggested to involve actors from the “[…] economy, [and] social sector[s]” (P42 – Research institute (Agriculture & Veterinary)) additional to actors on the human-animal-environment interface to get together in “[…] forums where scientists and policy-makers sit together to discuss the challenges they are facing” (P1 – University). This was also represented by the survey results, where none of the 104 respondents stated that communication between scientists and policy-makers on One Health issues is very easy, whereas 48% stated it to be difficult or very difficult (see Table 3). No statistically significant differences across regions (Nordic countries (*n* = 23), western (*n* = 48), southern (*n* = 16), eastern Europe (*n* = 11)) were detected [F(3, 72 = 0.569), *P* = 0.637].

**Table 3:** Respondents’ categorisation of communication and attention issues between scientists and policy-makers

	**Very difficult & difficult**	**Neither**	**Easy & very easy**
Communication between scientists and policy-makers on One Health issues	50 (48%)	40 (38%)	14 (13%)
	**Strongly disagree & disagree**	**Neither**	**Agree & strongly agree**
One Health receives adequate attention from policy-makers in my country	42 (40%)	26 (25%)	36 (35%)

AMR networks were exemplified by respondents as networks that work well. Mentioned were for example the Danish AMR surveillance programme (DANMAP) and the Joint Programming Initiative on Antimicrobial Resistance (JPIAMR). The JPIAMR is a “global collaborative organisation and platform”, and one of the respondents pointed out that “[t]here is a close connection between researchers and policy-makers in this field” (P91 – Funding institute) [14].

A factor that affects networks in each country uniquely are the established ministries and services under a country’s government. These may vary in number and types. For example, the Ministry of Health in Italy covers human and animal health, and the Ministry of Health and Social Affairs in Sweden covers human health – and not animal health – simultaneously to social welfare topics. Further, respondents pointed out that some countries like Belgium and Germany have a federal government structure, where powers are shared by the national and regional governments.

Additional to the structural aspects was a geographic perspective. Survey respondents from the 23 European countries represented on average fewer respondents from eastern European countries (1,25 respondents from 8 countries) with only one or two individuals representing their country (Table 1). On average, there were six respondents from four countries in the Nordic countries, six respondents from eight countries in western Europe and 5,3 respondents from three countries in southern Europe. Further, the response rate (RR) to the survey was lowest from the eastern European region (RR_Eastern Europe_= 22.2%; as compared to RR_Western Europe_= 55.4%, RR_Nordic countries_ = 53.3%, RR_Southern Europe_ = 43.2%).

### Relations of scientists and policy-makers

Respondents perception of whether One Health receives adequate attention from policy-makers in their respective country was more equally distributed with 40% of respondents strongly disagreeing or disagreeing, and 35% agreeing or strongly agreeing, see Table 3. No statistically significant differences across regions (Nordic countries, western, southern, eastern Europe) [F(3,72 = 0.569), *P* = 0.637] or educational backgrounds (social (*n* = 13) and natural sciences (*n* = 68)) [t(79) = 0.342, *P* = 0.733] were detected. The issue of receiving attention, as a respondent working with environmental themes at the WHO described, is that “One Health requires a long-term strategic approach and policy-makers generally take a short-term view” (P47 – WHO). To drive One Health policies forward, the interests and priorities of research institutes must align with those of politicians, as research institutes are “dependent on the willingness of politics” (P28 – ICARS). Many respondents emphasised that priorities of politicians might change after the end of an election cycle. Further, interest or priorities of policy-makers may have an incomplete focus. For example, a respondent lamented that the European Commission focuses “[…] too much on AMR in a One Health perspective” and misses “[…] the broader scope” (P31 – University).

In terms of relations between the source and receiver, perceptions of respondents on leadership for One Health highlighted challenges for associating knowledge. Respondents expressed the need for stronger leaders to bring together different sectors, push forward the One Health approach and implement governance structures. In the specific case of AMR, this appeared to be perceived as more successful. Many respondents mentioned established networks and initiatives for AMR (e.g. JPIAMR, DANMAP, EU action plan against AMR) and reasoned that policy-making for AMR works well.

Leaders can be identified within national and international institutions that were ranked in the survey according to the respondents’ perceived importance for driving One Health policies forward, see Table 4. Both on international and national level, the main explanations for the ranking by the respondents were the actors’ roles and influences. Internationally, the WHO, the Food and Agriculture Organization of the United Nations (FAO) and the WOAH were ranked to be within the five most important actors. The United Nations Environment Programme (UNEP) was not included in the ranking but highlighted by many respondents as important, because the UNEP is engaged with the WHO, the FAO and the WOAH, forming the Quadripartite who aim to tackle One Health issues. The respondents perceived their roles and influence as strong, describing the organisations as “trendsetters” (P33 – Ministry) who “take a lead globally” (P76 – Research institute (veterinary & food)).

**Table 4:** Respondents’ ranking of importance of international and national actors

Ranked international actors	Average importance^a^ (in descending order)	Ranked national actors	Average importance^b^ (in descending order)
1. WHO	3.95	1. Government agencies	1.54
2. EFSA	4.26	2. National research institutes	2.12
3. ECDC	4.27	3. Universities	2.98
4. WOAH	4.94	4. Regional research institutes	3.90
5. FAO	5.57	5. Local research institutes	4.46

^a^11 levels of importance. Other actors were: Med-Vet-Net Association (6.63); One Health Commission (6.78); One Health Initiative (6.96); International research institutes (7.34); One Health Platform (7.56); European Environmental Agency (EEA) (7.74)

^b^5 levels of importance

The EFSA and the European Centre for Disease Prevention and Control (ECDC) were placed on second and third place respectively, indicating their important roles. A Swedish respondent explained: “European One Health policies must be driven by the European institutions dealing with these matters together with the member states and their research institutions” (P19 – Veterinary institute). The ranking did not include the European Medicines Agency (EMA), but respondents emphasised the agency as an important actor. The only European agency that was deemed unimportant was the EEA. Another actor that was not listed in the ranking but mentioned by respondents was the European Commission. It was suggested that the Commission as a “central player”, could appoint a “[…] secretariat or commissioner” (P8 – Museum) to focus on One Health topics.

Nationally, the actors that ranked from highest to lowest importance were government agencies, national research institutes, universities, regional, local research institutes. The ranking did not take into account potential structural differences across countries, like federal structures in Austria, Belgium, Germany and Switzerland; or the lack of local and regional agencies, such as in France and Czech Republic.

In comparison to the international actors, the national actors were on average perceived to be more important for driving One Health policies forward (see average importance in Table 4). The respondents argued for the national actors’ importance by pointing out the role of research institutes and universities as influencing policy-making, and the role of government agencies as policy-makers. This was explained by two respondents who stated that government agencies “have the power to implement policies based on science and technical support from national research institutes” (P1 – University), and they “[…] can have direct input into national policy definition” (P25 – Research institute (Food & Agriculture)). Universities as well as local and regional agencies were seen to have some influence through their scientific and advisory contributions. One respondent emphasised the role of universities in the ranking, explaining that the education of the One Health approach potentially has future impact for One Health policies.

### Influencing factors

This section comprises influencing factors that can affect the knowledge translation process through different actors and aspects. An influencing factor that presented a challenge for implementing the One Health approach was identified in the survey as the different “languages” spoken in science and politics. Respondents labelled the scientific language as “technical”, “complex”, “detailed”, and “inferred” (P44 – Research institute (Veterinary, Environment & Food)); P7 – Food institute; P50 – N/A; P84 – Public health institute). On the other hand, the political language was described according to policy-makers needs of “simple statements that can be easily understood”, “concrete messages about what can be done”, and that policy-makers are “more interested in the bottom line and want straight forward answers” (P7 – food institute; P28 – ICARS; P50 – N/A). Accordingly, respondents identified the lack of training to communicate scientific findings to politicians, including the absence of a “compelling narrative” (P70 – WHO) as factors impairing to motivate One Health actions.

An additional challenge to the different “languages” across sectors were the different understandings of the One Health approach. 98% of respondents agreed or strongly agreed that they completely understand what One Health means. Yet, throughout the survey, respondents highlighted the “different meanings of One Health” (P53 – Public health institute). The capability of establishing a common understanding of the One Health approach remains a challenge. One respondent acknowledged that “there is no clear view of One Health” (P57 – Food institute), inhibiting translation to politics, supported by another statement that there is “limited understanding of the One Health approach by policy-makers” (P52 – WHO). Blamed for this was for example the complexity of the One Health approach with its intertwined relationships on the human-animal-environment interface (P56 – Public health institute). Further, there were concerns that One Health “has lost most of its meaning” (P96 – Ministry) and that it “is becoming a buzzword!” (P58 – Research institute (Agriculture, Environment & Food)), which might diminish importance and significance of the One Health approach.

## Discussion

The One Health approach is a global paradigm. However, the survey was geographically limited to Europe and perspectives of experts working within European institutes and agencies. Further, the lack of access to respondents from the social sciences, ecology, and economic sectors causes a narrower view on One Health that neglects environmental (including plant and ecological), societal and community efforts and issues. Main One Health topics addressed were zoonoses, AMR and food safety. It is important to highlight the manifold issues that One Health can address (e.g. behaviours [15], climate change [16], non-communicable diseases [17]), as they are essential for a comprehensive understanding of One Health.

Nevertheless, the study demonstrated the importance of connecting knowledge from scientists to policy-makers. The survey identified several challenges for knowledge association of One Health in terms of institutional barriers, and challenges of communicating scientific information to policy-makers. The challenges were structured in three sub-headings: (1) Leadership; (2) Political attention; (3) Languages and meanings. Table 5 shows the three dimensions (networks, relations and influencing factors), the corresponding challenges for the knowledge translation process, and potential solutions identified within the study.

**Table 5:** Dimensions of the knowledge translation process, challenges and potential solutions

	Challenges	Potential solutions
Networks	Lack of leadership	• Approach One Health issue individually (like AMR); • Engage eastern European experts into One Health networks; • Problematising to establish scope and target.
Relations	Lack of political attention	• Identify appropriate, valuable and tangible information for policy-makers; • Establishing strong relations with national actors; • Learning from successful activities (e.g. AMR); • Select leaders from NGOs and EU agencies.
Influencing factors	Lack of context	• Engage social, political and economic actors; • Determine meaning of One Health for each activity.
	No common language among scientists and policy-makers	• Glossary; • Communication training; • Employing communication experts.

### Leadership

Within networks, information can be shared about multifaceted One Health-related topics. However, survey respondents lamented the sparse collaboration across ministries, which indicates a lack of formal or informal networks. Good leadership is a way to establish and maintain networks that bridge across ministries, sectors and countries. The employment of One Health leaders is mentioned in the literature, referring to abilities of performing strategic analysis, finding solutions, organising, and employing flexible and transparent approaches [18, 19]. However, in relation to the complexity of One Health activities, more concrete characteristics of leaders must be discussed. The One Health approach is often implemented in scientific or administrative settings, where project managers or principal investigators are responsible for conducting projects and leading interdisciplinary teams. Literature on leadership often refers to leadership in organisations. Some aspects of this can apply or be adapted to the scientific context like research projects, and administrative contexts for coordinating interdisciplinary activities. Marion and Uhl-Bien [20] suggest that leaders must strengthen networks while being aware of their interdependencies and dynamics, as well as encourage them by facilitating communication. In the survey, communication between scientists and policy-makers was perceived as rather difficult across all regions. Interestingly, the response rate of the survey was lowest from the eastern European region. The limited participation of eastern European experts suggests less communication and fewer networks within those countries. Fewer One Health-related publications and a lack of co-citations of authors from eastern European countries also indicates sparse discussion of One Health on a political level and across scientists [21, 22]. Engaging eastern European experts into One Health networks can facilitate communication among scientists and between scientists and policy-makers. The notion of facilitating the role of networks is crucial as actors within those networks have “information about what the different government organizations with which they interact are doing” [23]. Combining this information can clarify the usefulness of activities, link it to needs and capacities, and enable cross-ministerial policy coordination [12]. Hence, within networks leaders can facilitate knowledge translation, and foster communication, collaboration and the sharing of information.

To make each One Health issue manageable, it is desirable to approach them individually, and clearly formulate tasks and scope of the project or activity [20]. For example, the latest report on the Danish AMR surveillance described that DANMAP was only made possible through some active scientists, advocating and taking the lead to establish the national surveillance and monitoring system [24]. However, other, less well-defined One Health issues must first be problematised to assess specific challenges within and across the sectors. While the DANMAP is comprehensive, acknowledging public and animal health issues, as well as some environmental aspects, it is important to note the lack of engagement of the environment sector [24, 25]. This underlines the importance of leadership able to problematise AMR, push it forward and implement it. On EU level, AMR is also a priority. This was exemplified by the European Commission’s support of the JPIAMR, which problematises the issue of AMR by defining key areas that need to be addressed, and providing leadership through coordination, guidance as well as resources. This has resulted in over hundred research projects and activities. Of course, the JPIAMR has a specific focus on AMR, with a stronger emphasis on issues from the medical, epidemiological and biological disciplines [14]. Nevertheless, examining those processes, from problematising AMR to developing policies, will provide lessons learned that can be applied to other One Health topics. The contextualisation of One Health issues for the receiver (e.g. actors within ministries) enables an understanding of the implications, as it establishes the usefulness of the activity via outlining tasks, roles and responsibilities.

### Political attention

Problematising One Health issues can also help to catch political attention. The survey displayed that many respondents disagreed that there is political attention on One Health due to politician’s periods in office that entail short-term agendas, as opposed to long-term approaches needed for successful One Health activities. The respondents’ perception of missing political attention was not statistically significant across regions or educational backgrounds (social or natural sciences). However, there were fewer respondents with social science backgrounds. This can indicate a lack of social scientists within One Health networks, highlighting the need to engage and involve those actors into the One Health approach. Social scientists can aid in catching or facilitating political attention by using social, economic or political arguments that can help to associate One Health issues with current politics [15, 26].

Capturing political attention can result in policy development as well as the allocation of funding [27]. The lack of funding for One Health-related activities was mentioned by the majority of respondents as a challenge for implementing the One Health approach and also corresponds with the literature [2]. However, an underlying challenge to the lack of funding is the translation of knowledge on an institutional and political level. Translation of knowledge across sectors, through collaboration, networks and good relations might be as, or even more important for implementing the One Health approach, as it is the prerequisite for receiving funding. To raise the attention of politicians regarding any One Health issue, it is crucial to associating knowledge by identifying valuable information that policy-makers can relate to and find tangible.

Productive relations between the source and receiver are crucial for knowledge translation, and are affected by the work environment, which ideally should be an environment of trust and openness to discussion [20]. Discussions become crucial to address different agendas, roles, priorities and interests among the actors, and how to align them [28]. National actors were perceived as very important for pushing One Health policies forward, especially government agencies and national research institutes. Establishing strong relations within those networks, as a fundament to translate, problematise and associate knowledge will facilitate the implementation of One Health activities.

There is no one-fit-for-all solution for catching political attention, as the allocation of services under ministries is different across states, and different government systems (like federal systems) affect how powers are distributed within a state [19]. Considering a governments structure is important for national One Health approaches, as it can facilitate but also impede the establishment of networks.

International actors, identified by the survey respondents who can catch political attention and drive One Health policies forward were the Quadripartite organisations. The Quadripartite did not conceive the One Health approach, but they adopted it as a cross-sector collaboration. Their aim has been to establish a coherent approach to tackle One Health issues [29, 30]. While the organisations approach is not perfect, for example due to little emphasise on plant health or engagement of society, the agencies are recognised as important actors, not least by the survey respondents [31, 32]. Among the Quadripartite organisations, the WHO was perceived as the most important actor to push One Health policies forward. This might reflect the WHO’s role as a global actor in tackling a broad range of health-related topics, including environmental factors and interdisciplinary topics like outbreaks and pandemics [33]. The EFSA, ECDC and EMA were also identified as important agencies due to their advising role to the European Commission who has the ability to propose and influence new EU laws and policies. Hence, the Quadripartite, especially the WHO, and the EU agencies were perceived to have power and influence through their positions, which they can use for One Health-related policy- and decision-making. The EEA was the only EU agency that was not considered to be an important actor to push One Health policies forward. A factor might be that the role of the EEA differs from those of the EFSA, ECDC and EMA. The latter three agencies have regulatory functions, while EEA’s function is consultative, focusing on networking and sharing information on practices as well as policies [34, 35]. The lack of perceived importance of the EEA on EU level can impede a comprehensive and interdisciplinary approach to One Health issues. Environmental and ecological considerations (including plants) are crucial for tackling One Health issues [25]. Regardless of the lack of regulatory functions, the EEA can promote the One Health approach by clarifying their role and being receptive or initiating to engage in collaboration for One Health activities.

### Languages and meanings

The understanding of what the One Health approach is varies among sectors and actors, and some survey respondents feared that it might lose meaning by becoming a buzzword or label instead of becoming a concurred approach, utilising the philosophy behind it and the tools it can provide. Determining the meaning and philosophy is important for a One Health activity as it facilitates defining scope and tasks [36, 37]. Creating value and meaning is crucial to prevent the occurrence of buzzwords - or confusion by creating yet another term [38]. It entails carefully considering the research or activity, evaluating if it is in fact “One Health” or if it does not concern all items on the human-animal-environment interface. This might result in different meanings of the One Health approach in different contexts.

Contextualising One Health issues can help to design, implement and raise awareness of One Health activities. This means to understand decision-making processes and provide societal perspectives [39]. For this, actors with social, political and economic backgrounds are well equipped [26]. These actors are underrepresented within the One Health approach, as mentioned by survey respondents and in the literature [15, 21, 26, 40, 41]. The inclusion of social, economic and political scientists into One Health networks can accumulate new perspectives on how to tackle complex issues, for example the potential of gender-responsive perspectives to consider health disparities, glocal governance approaches, or by providing methods that allow gathering context dependent data or data relying on cultural knowledge [15, 42, 43]. This can help to illustrate and contextualise implications and provide insight into societal aspects that can benefit the creation of One Health activities [26, 40].

Further factors that influence knowledge translation were the capabilities and skills of scientists to construct and communicate a “compelling narrative” (P70 - WHO) for One Health issues to spark interests of other scientists to engage in collaboration, and to spark the interest of policy-makers. For knowledge translation among scientists of different disciplines, existing tools such as glossaries can facilitate a common language (e.g. https://foodrisklabs.bfr.bund.de/ohejp-glossary/ [44]). The interdisciplinary nature of the One Health approach makes it especially difficult to break down issues to an understandable and tangible form. It can be beneficial for scientists to have some communication background or training [45, 46]. Employing communication experts can be an option to promote knowledge translation from scientists to policy-makers, preventing misunderstandings or simply a disregard of the issue, and enhancing political attention and awareness of One Health topics.

## Conclusion

Implementing One Health activities is complex and relies on the commitment of actors across disciplines and sectors. To implement those activities, it is crucial to understand different aspects of the knowledge translation process. This study provided insight into this process from a European perspective, which can help to understand scientists and policy-makers’ relations, networks and some influencing factors. It highlighted the importance of knowledge translation by pointing towards challenges relating to leadership, political attention, meanings and understanding of “languages” within the One Health approach.

The study showed a lack of leadership, which impairs networks engaged in One Health activities. Establishing leadership that facilitates networks, also with and within eastern European regions where there are fewer, is likely beneficial to promote the One Health approach generally. Challenges also regard the relations among different actors on national and international level, which can lead to a lack of political attention for the One Health approach. Further, the influencing factors highlight issues with different understandings of One Health and a lack of context when implementing One Health activities. More engagement of social, political and economic actors could counteract this. As there are many disciplines and actors involved, finding a common language, promoting research communication capabilities and skills of scientists can provide a valuable tool to reach policy-makers and facilitate more attention to One Health topics.

To strengthen the implementation of One Health activities, future research could illuminate the role of other steps within the knowledge transfer model, such as awareness and acquisition as prerequisite to transforming knowledge.

## Supplementary Information

**Additional file 1.** “One Health governance” online survey questionnaire.

## Data Availability

The questionnaire of the online survey is available as supplementary information.

## Abbreviations

AMR: Antimicrobial Resistance
DANMAP: Danish Integrated Antimicrobial Resistance Monitoring and Research Programme
ECDC: European Centre for Disease Prevention and Control
EEA: European Environment Agency
EFSA: European Food and Safety Authority
EMA: European Medicines Agency
EU: European Union
FAO: Food and Agriculture Organization of the United Nations
ICARS: International Centre for Antimicrobial Resistance Solutions
JPIAMR: Joint Programming Initiative on Antimicrobial Resistance
OHEJP: One Health European Joint Programme
P: Participant
RR: Response rate
UNEP: United Nations Environment Programme
WHO: World Health Organization
WOAH: World Organisation for Animal Health (formerly known as OiE)

## References

[1] Adisasmito WB, Almuhairi S, Behravesh CB, Bilivogui P, Bukachi SA, One Health High-Level Expert Panel (2022). One Health: A new definition for a sustainable and healthy future. PLOS Pathogens..

[2] Regeer BJ, dos S. Ribeiro C, van de Burgwal LHM (2019). Overcoming challenges for designing and implementing the One Health approach: A systematic review of the literature. One Health..

[3] Lebov J, Grieger K, Womack D, Zaccaro D, Whitehead N, Kowalcyk B (2017). A framework for one health research. One Health..

[4] Novossiolova TA, Whitby S, Dando M, Pearson GS (2021). The vital importance of a web of prevention for effective biosafety and biosecurity in the twenty-first century. One Health Outlook.

[5] Hunter DJ (2016). Evidence-informed policy: in praise of politics and political science. Public health panorama.

[6] Ward V, Smith S, House A, Hamer S (2012). Exploring knowledge exchange: A useful framework for practice and policy. Soc Sci Med..

[7] Connolly J (2017). Governing towards ‘One health’: establishing knowledge integration in Global Health Security Governance. Global Policy.

[8] Hitziger M, Esposito R, Canali M, Aragrande M, Häsler B, Rüegg SR (2018). Knowledge integration in one health policy formulation, implementation and evaluation. Bull World Health Organ.

[9] Conrad PA, Meek LA, Dumit J (2013). Operationalizing a One Health approach to global health challenges. Comparative Immunol Microbiol Infect Dis..

[10] Straus SE, Tetroe J, Graham I (2009). Defining knowledge translation. CMAJ..

[11] Liyanage C, Elhag T, Ballal T, Li Q (2009). Knowledge communication and translation – a knowledge transfer model. J Knowledge Manage..

[12] Lavis JN, Ross SE, Hurley JE (2002). Examining the role of Health Services Research in Public Policymaking. Milbank Q.

[13] Rhodes RAW. How to Manage Your Policy Network. Vol. 1. United Kingdom: Oxford University Press; 2017 (Cited 1 Jul 2021]. Available from: https://oxford.universitypressscholarship.com/view/10.1093/oso/9780198786108.001.0001/oso-9780198786108-chapter-5.

[14] van Hengel AJ, Marin L (2019). Research, Innovation, and Policy: An Alliance Combating Antimicrobial Resistance. Trends in Microbiology..

[15] Saylors K, Wolking DJ, Hagan E, Martinez S, Francisco L, Euren J (2021). Socializing one health: an innovative strategy to investigate social and behavioral risks of emerging viral threats. One Health Outlook.

[16] Zinsstag J, Crump L, Schelling E, Hattendorf J, Maidane YO, Ali KO, et al. Climate change and One Health. FEMS Microbiol Lett. 2018 (Cited 9 Nov 2020 );365(11). Available from: https://www.ncbi.nlm.nih.gov/pmc/articles/PMC5963300/. 10.1093/femsle/fny085 PMC5963300 29790983

[17] Natterson-Horowitz B, Desmarchelier M, Winkler AS, Carabin H. Beyond Zoonoses in One Health: Non-communicable Diseases Across the Animal Kingdom. Frontiers in Public Health. 2022 (Cited 9 Aug 2022 );9. Available from: https://www.frontiersin.org/articles/10.3389/fpubh.2021.807186. 10.3389/fpubh.2021.807186 PMC8846287 35178374

[18] Stephen C, Stemshorn B (2016). Leadership, governance and partnerships are essential one health competencies. One Health..

[19] Kelly TR, Machalaba C, Karesh WB, Crook PZ, Gilardi K, Nziza J (2020). Implementing one Health approaches to confront emerging and re-emerging zoonotic disease threats: lessons from PREDICT. One Health Outlook.

[20] Marion R, Uhl-Bien M (2001). Leadership in complex organizations. Leadership Quarterly..

[21] Humboldt-Dachroeden S, Rubin O, Sylvester Frid-Nielsen S (2020). The state of one health research across disciplines and sectors – a bibliometric analysis. One Health.

[22] Sikkema R, Koopmans M (2016). One Health training and research activities in Western Europe. Infect Ecol Epidemiol..

[23] Peters BG (2018). The challenge of policy coordination. Policy Design and Practice..

[24] Attauabi M, Borck Høg B, Müller-Pebody B DANMAP 2020 - Use of antimicrobial agents and occurrence of antimicrobial resistance in bacteria from food animals, food and humans in Denmark. Copenhagen, Denmark: National Food Institute; Statens Serum Institut; 2020 (Cited 2 Nov 2021). Available from: https://www.danmap.org/reports/2020.

[25] Humboldt-Dachroeden S, Mantovani A (2021). Assessing environmental factors within the One Health Approach. Medicina.

[26] Lapinski MK, Funk JA, Moccia LT (2015). Recommendations for the role of social science research in one health 1. Soc Sci Med..

[27] Mortensen PB (2010). Political attention and public policy: a study of how agenda setting matters. Scandinavian Political Studies.

[28] Enroth H. Policy Network Theory. In: The SAGE Handbook of Governance [Internet]. London: SAGE Publications Ltd; 2011 (Cited 22 Jun 2021 ). p. 19–35. Available from: http://sk.sagepub.com/reference/hdbk_governance/n2.xml.

[29] FAO. OiE. WHO. The FAO-OIE-WHO Collaboration - Sharing responsibilities and coordinating global activities to address health risks at the animal-human-ecosystems interfaces - A Tripartite Concept Note [Internet]. The Food and Agriculture Organization of the United Nations; The World Organisation for Animal Health; The World Health Organization; 2010 (Cited 25 Feb 2022 ). Available from: https://www.oie.int/fileadmin/Home/eng/Current_Scientific_Issues/docs/pdf/FINAL_CONCEPT_NOTE_Hanoi.pdf.

[30] Villanueva-Cabezas JP. One health: A brief appraisal of the Tripartite – UNEP definition. Transboundary and Emerging Diseases. 2022 (Cited 19 Jul 2022 ); Available from: https://onlinelibrary.wiley.com/doi/abs/10.1111/tbed.14567. 10.1111/tbed.14567 35460594

[31] Andrivon D, Montarry J, Fournet S (2021). Plant Health in a one health world: missing links and hidden treasures. Plant Pathol.

[32] Hitziger M, Berezowski J, Dürr S, Falzon LC, Léchenne M, Lushasi K, et al. System thinking and Citizen participation is still missing in one Health Initiatives – Lessons from fifteen evaluations. Front Public Health. 2021;9:653398. 10.3389/fpubh.2021.653398 PMC8211880 34150701

[33] WHO. World Health Statistics. 2021: monitoring health for the SDGs, sustainable development goals. Geneva, Switzerland: World Health Organization; 2021 (Cited 19 Nov 2021). Available from: https://apps.who.int/iris/bitstream/handle/10665/342703/9789240027053-eng.pdf.

[34] Chatzopoulou S. The EU Agencies’ Role in Policy Diffusion Beyond the EU: EEA, EMA and EFSA [Internet]. Rochester, NY: Social Science Research Network; 2018 (Cited 1 Dec 2021). Report No.: ID 3105894. Available from: https://papers.ssrn.com/abstract=3105894.

[35] Wood M (2018). Mapping EU agencies as political entrepreneurs. Eur J Polit Res.

[36] Lerner H, Berg C. The concept of health in One Health and some practical implications for research and education: what is One Health? Infect Ecol Epidemiol. 2015 (Cited 10 Dec 2020);5. Available from: https://www.ncbi.nlm.nih.gov/pmc/articles/PMC4320999/. 10.3402/iee.v5.25300 PMC4320999 25660757

[37] Coghlan S, Coghlan BJ, Capon A, Singer P (2021). A bolder one health: expanding the moral circle to optimize health for all. One Health Outlook.

[38] Cornwall A, Brock K (2005). What do Buzzwords do for Development Policy? A critical look at ‘Participation’, ‘Empowerment’ and ‘Poverty reduction’. Third World Quarterly.

[39] Straus SE, Tetroe JM, Graham ID (2011). Knowledge translation is the use of knowledge in health care decision making. J Clin Epidemiol.

[40] Craddock S, Hinchliffe S (2015). One world, one health? Social science engagements with the one health agenda. Soc Sci Med.

[41] Degeling C, Rock M. Qualitative Research for One Health: From Methodological Principles to Impactful Applications. Front Vet Sci. 2020 (Cited 26 Feb 2020 );7(70). Available from: https://www.frontiersin.org/articles/10.3389/fvets.2020.00070/full. 10.3389/fvets.2020.00070 PMC7039926 32133378

[42] Garnier J, Savic S, Boriani E, Bagnol B, Häsler B, Kock R (2020). Helping to heal nature and ourselves through human-rights-based and gender-responsive one health. One Health Outlook.

[43] Rubin O (2019). The glocalization of antimicrobial stewardship. Globalization and Health..

[44] Buschhardt T, Günther T, Skjerdal T, Torpdahl M, Gehtmann J, Filippitzi ME (2021). A one health glossary to support communication and information exchange between the human health, animal health and food safety sectors. One Health.

[45] Mateus TL, Teixeira P, Maia RL. We Know One Health, but We also Need One Communication. In: Leal Filho W, Vidal DG, Dinis MAP, Dias RC, editors. Sustainable Policies and Practices in Energy, Environment and Health Research: Addressing Cross-cutting Issues. Cham: Springer International Publishing; 2022 (Cited 11 Feb 2022 ). p. 245–59. (World Sustainability Series). Available from: 10.1007/978-3-030-86304-3_15.

[46] Miller JD (2004). Public Understanding of, and Attitudes toward, Scientific Research: What We Know and What We Need to Know. Public Underst Sci..

