# Supplementary material for: Translating One Health knowledge across different institutional and political contexts in Europe
Source: One Health Outlook. 2023 Jan 31;5:1. doi: 10.1186/s42522-022-00074-x (PMC9890731; doi:10.1186/s42522-022-00074-x)
Supplement: Supplementary file 1 — Additional file 1. “One Health governance” online survey questionnaire. [file 42522_2022_74_MOESM1_ESM.docx]

Additional file 1 – “One Health governance” Online survey questionnaire

| Survey questions* | Response options |
| --- | --- |
| **Demographics** | |
| 1. What is your educational background? | Free text |
| 1. What is your job title? | Free text |
| 1. In what country do you mainly work? | Free text |
| 1. What is your workplace? | Free text |
| **Experiences with One Health** | |
| On a scale from 1 – 5, how would you rate your understanding of the One Health approach. 1: I do not understand what One Health is.  5: I completely understand what One Health is. | \| 1 \| 2 \| 3 \| 4 \| 5 \| \| --- \| --- \| --- \| --- \| --- \| \| 🔾 \| 🔾 \| 🔾 \| 🔾 \| 🔾 \| |
| - 1. Please elaborate in a few words how you understand One Health. | Open ended |
| 1. To what extent do you agree or disagree with the following statements? | \|  \| Strongly disagree \| Disagree \| Neither agree nor disagree \| Agree \| Strongly agree \| \| --- \| --- \| --- \| --- \| --- \| --- \| \| Covid-19 is a One Health issue. \|  \|  \|  \|  \|  \| \| Climate change is a One Health issue. \|  \|  \|  \|  \|  \| \| Biodiversity is a One Health issue. \|  \|  \|  \|  \|  \| \| Environmental contamination is a One Health issue. \|  \|  \|  \|  \|  \| |
| 1. Which area or areas do you work with? | - Zoonotic diseases - Antimicrobial Resistance - Food safety - Food security - Disease prevention and preparedness - Disease surveillance - Climate change - Environmental contamination - Biodiversity - Other, please specify |
| 1. What sectors are you in contact with when working with One Health issues? | - Public health - Human medicine - Veterinary science - Environment - Food - Political sector - I do not collaborate with any sectors - Other, please specify |
| **Science to Policy** | |
| 1. Who are the most important international actors when it comes to driving One Health policies forward? Please rank according to importance 1= Most important; 10 = Least importance | - World Health Organisation - Food and Agriculture Organization - World Organisation for Animal Health - Med-Vet-Net Association - One Health Commission - One Health Initiative - One Health Platform - EFSA - ECDC - International research institutes   Text box: Other, please explain your reasoning |
| 1. Who are the most important national actors when it comes to driving One Health policies forward? Please rank according to importance 1= Most important; 5 = Least importance | - Governmental agencies - National research institutes - Regional research institutes - Local research institutes - Universities   Text box: Other, please explain your reasoning |
| 1. To what extent do you agree or disagree with the following statements? | \|  \| Strongly disagree \| Disagree \| Neither agree nor disagree \| Agree \| Strongly agree \| \| --- \| --- \| --- \| --- \| --- \| --- \| \| One Health receives adequate attention from policy-makers in my country. \|  \|  \|  \|  \|  \| \| Science to policy translation for One Health issues is successful. \|  \|  \|  \|  \|  \| |
| - 1. If you Strongly agree or Strongly disagree that science to policy translation for One Health issues is successful, please elaborate why. | Open ended |
| 1. How would you categorise communication between scientists and policy-makers on One Health issues? | \| Very easy \| Easy \| Neither easy nor difficult \| Difficult \| Very difficult \| \| --- \| --- \| --- \| --- \| --- \| \| 🔾 \| 🔾 \| 🔾 \| 🔾 \| 🔾 \| |
| - 1. If Difficult or Very difficult: What are the main barriers for communication between scientists and policy-makers? | Open ended |
| **Coordination of One Health** | |
| 1. What initiatives, projects or programmes are you aware of in your country that focus on One Health? | \|  \| I am aware of, please provide an example \| I am not aware of \| \| --- \| --- \| --- \| \| Initiatives, projects or programmes on national level \| 🔾 \| 🔾 \| \| Initiatives, projects or programmes on regional level \| 🔾 \| 🔾 \| \| Initiatives, projects or programmes on local level \| 🔾 \| 🔾 \| \| Initiatives, projects or programmes on international level \| 🔾 \| 🔾 \| |
| 1. What are the three main challenges in your country for the implementation of One Health? | - Lack of communication between institutions - Lack of collaboration between institutions - Lack of collaboration between ministries - Confusing legislations - Lack of guidance - Lack of education and training - Lack of willingness - Lack of funding - Lack of political awareness - Inadequate governance/leadership - Other, please specify |
| How do you think the public should be informed about One Health? (Choose the three most important outlets) | - Social media - Print media - TV news - Campaigns - Public meetings - Websites - Radio - Education - Other, please specify |
| 1. Do you think informing the public about One Health will lead to more discussions about One Health on a political level? | - Yes - No - Don’t know |
| **End** | |
| Thank you very much for participating in the survey! Do you have any additional comments regarding coordination and implementation of One Health? | Free text |

*The questionnaire also contains the section “Coordination of antimicrobial resistance activities”. This section was not used in the article and was therefore omitted.
